# Characterization of the most frequent ATP7B mutation causing Wilson disease in hepatocytes from patient induced pluripotent stem cells

**DOI:** 10.1038/s41598-018-24717-0

**Published:** 2018-04-19

**Authors:** Silvia Parisi, Elena V. Polishchuk, Simona Allocca, Michela Ciano, Anna Musto, Maria Gallo, Lucia Perone, Giusy Ranucci, Raffaele Iorio, Roman S. Polishchuk, Stefano Bonatti

**Affiliations:** 1 Department of Molecular Medicine and Medical Biotechnology, University of Naples Federico II, Naples, Italy; 2 Department of Translational Medical Science, Section of Pediatric, University of Naples Federico II, Naples, Italy; 3 Telethon Institute of Genetics and Medicine, Pozzuoli, Italy

## Abstract

H1069Q substitution represents the most frequent mutation of the copper transporter ATP7B causing Wilson disease in Caucasian population. ATP7B localizes to the Golgi complex in hepatocytes but moves in response to copper overload to the endo-lysosomal compartment to support copper excretion via bile canaliculi. In heterologous or hepatoma-derived cell lines, overexpressed ATP7B-H1069Q is strongly retained in the ER and fails to move to the post-Golgi sites, resulting in toxic copper accumulation. However, this pathogenic mechanism has never been tested in patients’ hepatocytes, while animal models recapitulating this form of WD are still lacking. To reach this goal, we have reprogrammed skin fibroblasts of homozygous ATP7B-H1069Q patients into induced pluripotent stem cells and differentiated them into hepatocyte-like cells. Surprisingly, in HLCs we found one third of ATP7B-H1069Q localized in the Golgi complex and able to move to the endo-lysosomal compartment upon copper stimulation. However, despite normal mRNA levels, the expression of the mutant protein was only 20% compared to the control because of endoplasmic reticulum-associated degradation. These results pinpoint rapid degradation as the major cause for loss of ATP7B function in H1069Q patients, and thus as the primary target for designing therapeutic strategies to rescue ATP7B-H1069Q function.

## Introduction

Wilson disease (WD) belongs to the group of the most frequent inherited liver disorders. Its incidence is evaluated in the range of 1:7000 birth cases^1,2^. WD is caused by mutation in the efflux copper transporter ATP7B which leads to toxic accumulation of copper in the liver and subsequently in the brain with development of severe hepatic, neurological and psychiatric symptoms^3–5^. Existing WD treatments either target copper adsorption in the intestine or promote urinary excretion of excess metal^3–5^. Although helpful, these treatments cause serious side effects and exhibit limited efficacy in substantial cohorts of patients^6,7^. Thus, alternative therapeutic approaches are highly desirable.

While a great number of mutations have been reported to hamper the function of ATP7B, the majority of Caucasian patients (over 50%) harbors the H1069Q mutation either in homozygosity or compound heterozygosity^8^. The molecular mechanisms underlying the effects of the H1069Q mutation has been studied overexpressing the protein in heterologous or hepatoma-derived cell lines^9–13^. The wild-type transporter is located in the trans-Golgi network (TGN) where it pumps Cu ions from the cytosol into the lumen where this metal is bound by protein acceptors like ceruloplasmin^14,15^. In the presence of excess copper, wild type ATP7B reaches the endo-lysosomal compartment and the plasma membrane of bile canaliculi to guarantee copper excretion through the bile^16,17^. In contrast, the overexpressed mutant protein accumulates in the endoplasmic reticulum (ER), does not reach the Golgi and therefore does not mobilize in response to copper overload. Interestingly, although this mutation affects the nucleotide binding domain of the protein, it leaves significant levels of residual transport activity^11,18^ which however is useless (and most likely harmful) when the transporter cannot traffic between Golgi and endo-lysosomes. Unfortunately, animal models for this form of WD have not been generated yet. Recently the H/Q substitution was achieved in drosophila ATP7 (dATP7)^19^. However, insects lack liver and dATP7 functions resemble those of mammalian ATP7A rather than ATP7B because dATP7 suppression leads to a severe Cu deficiency similar to Menkes syndrome in humans^20^. Furthermore, hepatocytes from liver biopsies do not represent a realistic option due to the scarce amount of cells in a biopsy and the inability of hepatocytes to proliferate *in vitro*. Another possibility consists in gene editing of primary hepatocytes. However, despite a recent advance of Clustered Regularly Interspaced Short Palindromic Repeats-associated protein-9 nuclease (CRISPR-CAS9) technology, the probability to generate point mutation in both *ATP7B* alleles in substantial number of primary cells remains extremely low^21^.

Thus, the view summarized above has not been confirmed yet in a proper H1069Q cell system. However, we and others have provided proof of principle for new correction strategies to contrast the disease: different treatments such as incubation at low temperature or with curcumin^9,11^, expression of CRYAB^12^, inhibition of p38 and JNK kinase^13^, were all effective in rescuing the localization and/or Cu response of the overexpressed mutant.

The generation of induced pluripotent stem cells (iPSCs) has provided a revolutionary way to study and model human diseases and to overcome the limitations of non-isogenic cell models^22^. Thus, we have generated human iPSCs from primary fibroblasts of homozygous H1069Q WD patients and their closest familiar control available^23^. Here we present the characterization of these iPSCs, their differentiation toward hepatocyte-like cells (HLCs) and the results obtained from the analysis of the expressed ATP7B forms. Most surprisingly, we found that one third of the endogenously expressed H1069Q mutant was in the Golgi complex and moved to the endo/lysosomal compartments in response to Cu overload. However, the transporter exhibited a faster turnover in patient HLCs and consequently accumulated at a much lower level at steady state than in control HLCs, indicating rapid degradation of newly-synthetized protein as a major cause for the loss of function in H1069Q patients.

## Results

### Generation, characterization and hepatic differentiation of WD and control hiPSCs

**Figure 1:**
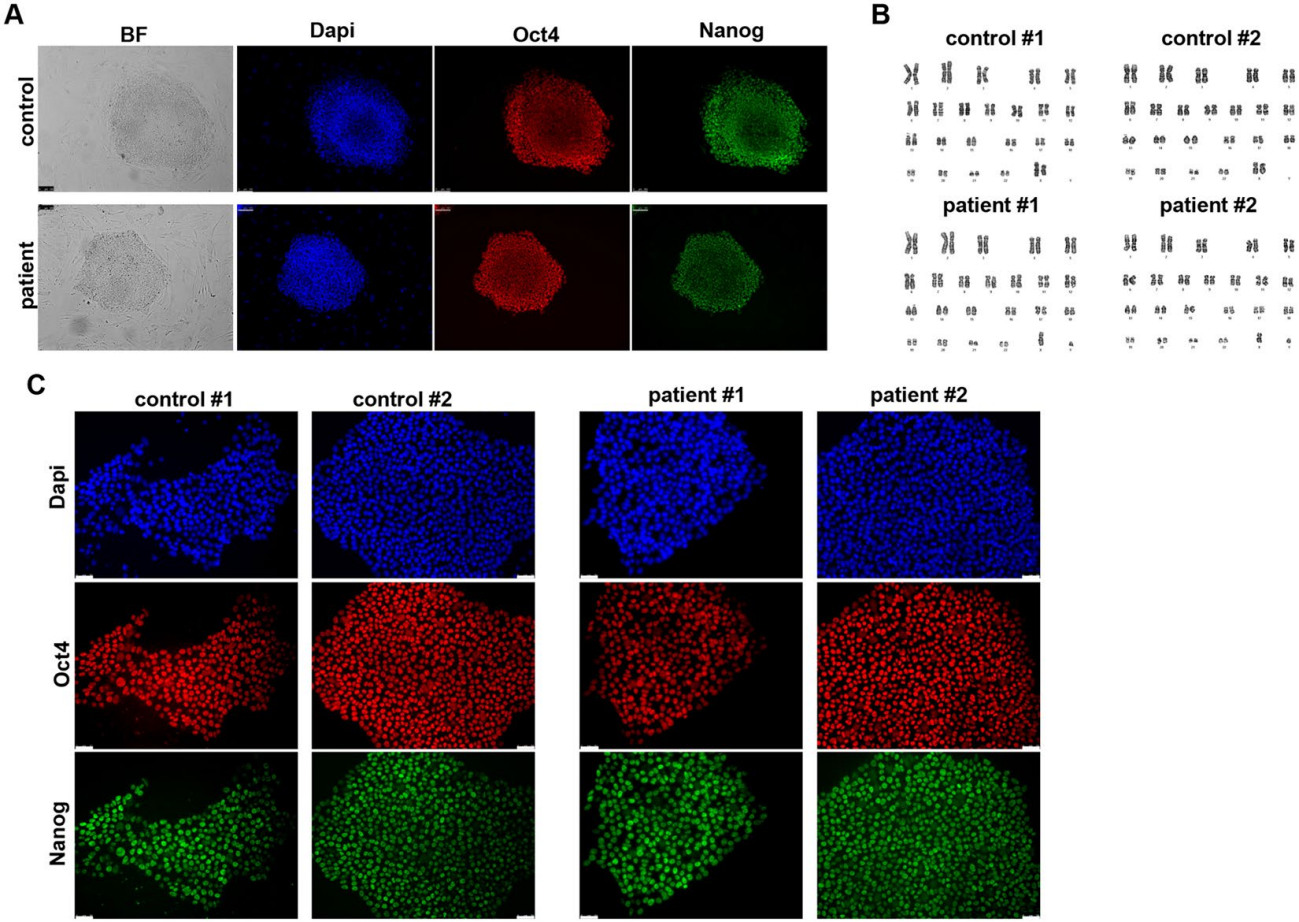
Generation of iPSCs from human fibroblasts heterozygous and homozygous for the H1069Q mutation of ATP7B. (**A**) Human fibroblasts from control (WT/H1069Q) and patient (H1069Q/H1069Q) were induced to reprogram and after 21 days the presence of pluripotent stem cell colonies was assessed by immunofluorescence for expression of the stemness markers Oct4 and Nanog. Nuclei are stained with DAPI. Scale bars: 100 µm. (**B**) Karyotype analysis of two clones of control and patient iPSCs showing no detectable aberration in chromosomes number and structure. (**C**) Two selected clones of control and patient iPSC were expanded and adapted to grow without feeder layer for at least 5 passages. The maintenance of the undifferentiated phenotype was assessed by immunofluorescence for stemness marker expression as in panel A. Scale bars: 50 µm.

To generate iPSCs we obtained primary fibroblasts from skin biopsies of a WD patient carrying the H1069Q mutation (H1069Q/H1069Q, see supplemental material, patient # 1) and his mother as a control (WT/H1069Q) (henceforth referred to as patient and control). iPSCs were generated using an integration-free method by transfecting episomal plasmid vectors into primary fibroblasts^24^, thus avoiding genetic integration in the reprogrammed cells. iPSC-like colonies emerged three weeks after transfection and their stemness was assessed by immunofluorescence for Nanog and Oct4 positivity (Fig. 1A). Two clones of each WD and control iPSCs were subjected to cytological analysis and showed a normal karyotype (Fig. 1B). These clones were adapted to grow in feeder-free conditions to avoid feeder contamination during differentiation and then analyzed for the maintenance of stemness after multiple passages (≥15). The expression of pluripotent stem cell marker, Oct4 and Nanog, was assessed by immunofluorescence microscopy and quantitative real-time PCR analysis (Fig. 1C and Supplementary Fig. S1). In addition, the pluripotent properties of the iPSC clones were evaluated by differentiating them into derivatives of the three germ layers (Supplementary Fig. S1).

**Figure 2:**
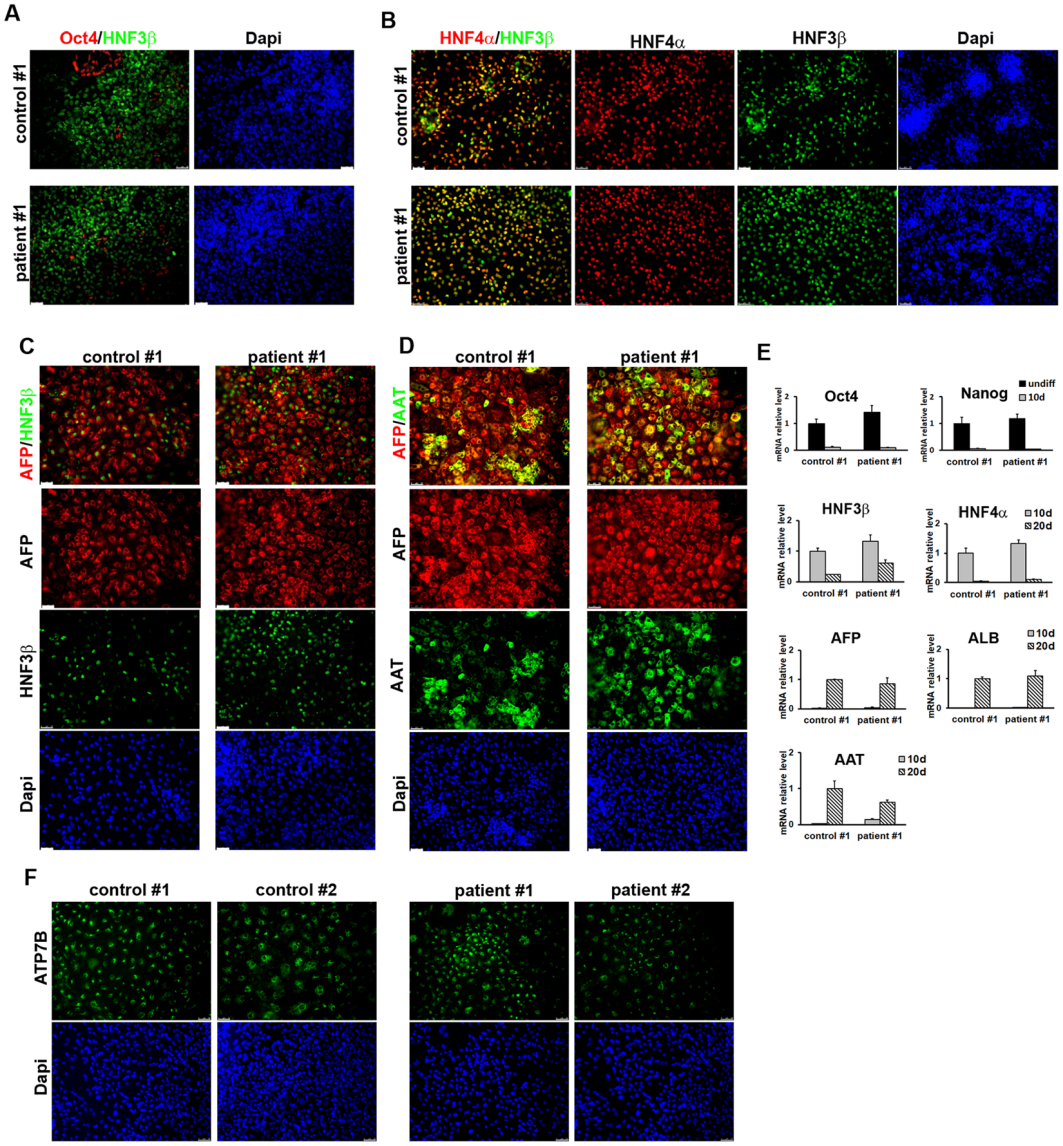
Characterization of HLCs obtained from control and patient iPSC clones. (**A**) One control and one patient iPSC clones shown in Fig. 1C were induced to differentiate into HLCs and after 5 days the achievement of endodermal phenotype was measured by immunofluorescence to visualize HNF3β protein expression. The parallel loss of the undifferentiated phenotype is proved by the disappearance of Oct4 positive cells. (**B**) After 10 days from HLC induction, hepatic specification of endodermal cells is reached by both control and patient cells as demonstrated by the high number of cells co-expressing HNF4α (hepatic progenitor marker) and HNF3β. (**C**) The differentiation of control and patient cells into the hepatic lineage was assessed at 15 days of HLC induction by immunofluorescence for the expression of the markers AFP and HNF3β. (**D**) Full hepatic differentiation was evaluated at 20 days by analyzing the presence of the functional hepatic marker AAT co-expressed with AFP. Scale bars: 50 µm. (**E**) the differentiation into HLCs was evaluated at the indicated time points by quantitative real-time PCR measuring the loss of the undifferentiated phenotype (Oct4 and Nanog) and the appearance of markers of the three hepatic developmental stages: definitive endoderm (HNF3β), hepatic progenitors (HNF3β, HNF4α, AFP) and hepatic-like cells (AFP, AAT). The graphs represent the mRNA relative expression of each marker normalized to the control clone levels. Data are reported as means ± SEM of at least three biological replicates. (**F**) Immunofluorescence analysis of the expression of ATP7B in HLCs derived from control and patient iPSCs. Scale bars: 50 µm.

Next, we differentiated patient and control iPSCs into HLCs using a previously established protocol^25^ that mimics three developmental stages of liver development: definitive endoderm, hepatic progenitors and hepatocyte-like cells. The expression of markers corresponding to the three phases was verified by immunofluorescence microscopy and quantitative real-time PCR. By 5 days of differentiation most cells expressed the endodermal marker hepatocyte nuclear factor 3β (HNF3β) (84.5% ± 5.2) with a concomitant disappearance of the stem cells marker Oct4 (Fig. 2A and Supplementary Fig. S2). The adoption of the endodermal phenotype was complete at 10 days of differentiation (Fig. 2B and Supplementary Fig. S2), when the differentiated iPSCs start to express the hepatic progenitor markers hepatocyte nuclear factor 4α (HNF4α) and α-fetoprotein (AFP). At 15 days of differentiation, positive immunostaining for AFP co-expressed with HNF3β confirmed the efficient differentiation of endoderm into hepatic lineage (Fig. 2C and Supplementary Fig. S2). The cells were further differentiated toward the hepatic lineage until 20 days when they resulted positive for albumin (ALB) and α1-antitrypsin (AAT), markers of more differentiated hepatic cells (Fig. 2D and Supplementary Fig. S2). Efficient hepatic differentiation *in vitro* was confirmed by measuring the number of cells co-expressing the hepatic marker AFP and the hepatic functional gene AAT (27.6% ± 8.6) after the final maturation phase (Fig. 2D and Supplementary Fig. S2). Importantly, no statistically significant differences were observed in the expression of differentiation markers between the patients and controls iPSC clones, indicating that ATP7B mutation does not impair hepatic differentiation in the conditions used. Therefore, we interchangeably used these clones for further analysis. Furthermore, we have fully reproduced the generation of iPSCs and their differentiation in HLCs starting from primary fibroblasts obtained from another homozygous H1069Q patient and his closest familiar control available (wt/H1069Q) (Supplementary Fig. S3) and henceforth referred to as 2nd patient and 2nd control.

### ATP7B expression in HLCs from patient and control iPSCs

We analyzed the expression of ATP7B in HLCs derived from patient and control iPSCs in all differentiation experiments by immunofluorescence and found that both in control and patient HLCs 30–40% of the total cell population was positive for the protein (Fig. 2F). Furthermore, quantitative real-time PCR data indicated that ATP7B expression is strongly induced during hepatic differentiation starting at 10 days of differentiation at comparable levels in HLCs from patient and control iPSCs (Supplementary Fig. S4). To rule out possible DNA rearrangements, the presence of the mutation in the ATP7B mRNA form was confirmed by amplifying and sequencing the region across the mutation from patient and control HLCs (Supplementary Fig. S4). Of note, the hepatic marker AAT was detected in 72 ± 23% and 77 ± 21% of ATP7B-positive HLCs from the control and patient clones respectively (Supplementary Fig. S4). All these data support the use of HLCs from control and patient iPSCs as a powerful isogenic *in vitro* model of WD to study the localization and activity of the H1069Q ATP7B mutant.

### A substantial amount of ATP7B localizes in the Golgi of patient HLCs

**Figure 3:**
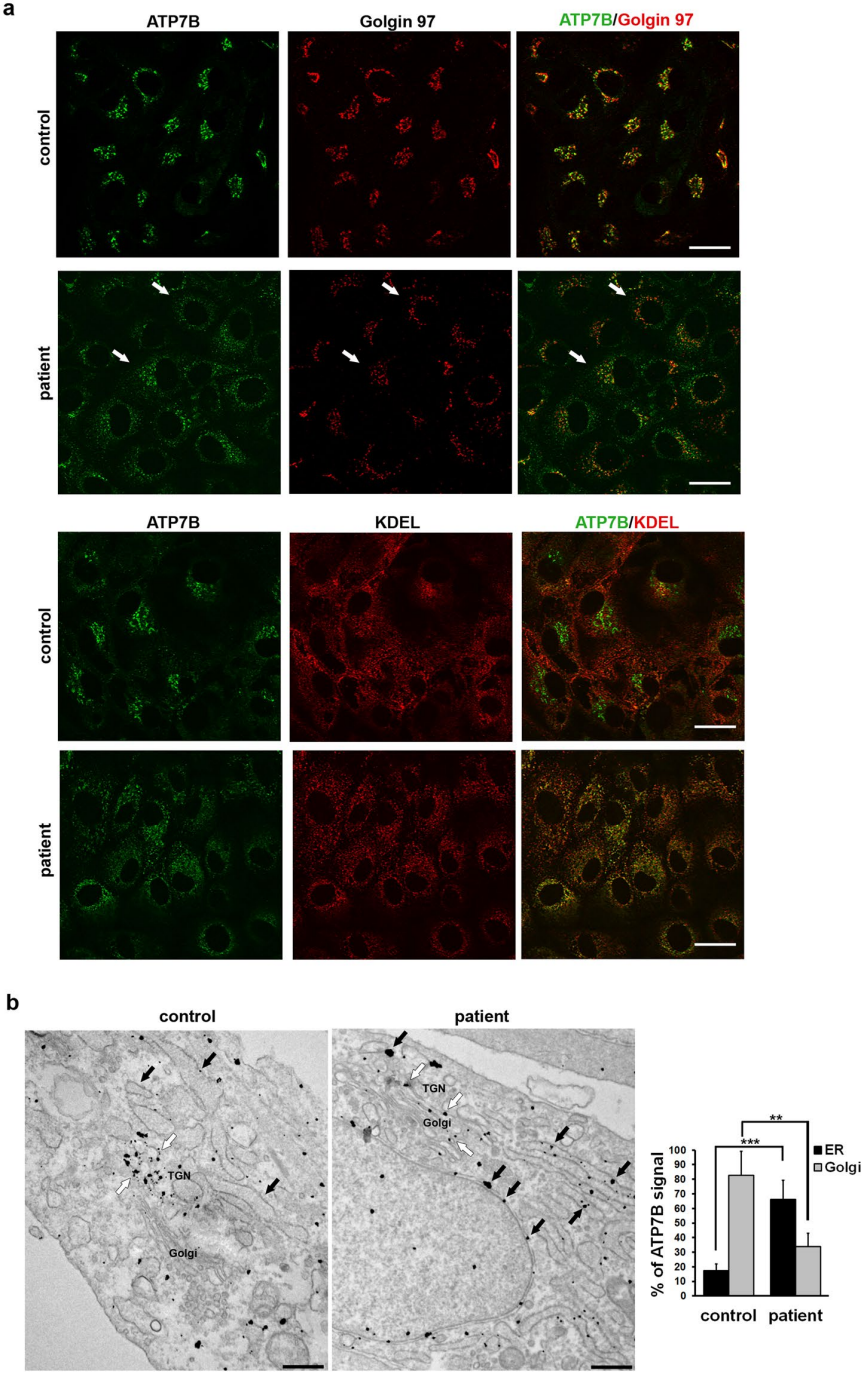
Intracellular localization of ATP7B in HLCs obtained from control and patient iPSCs. (**a**) Confocal immunofluorescence analysis of HLCs immunolabeled for ATP7B and Golgin 97 (upper panels) or KDEL containing proteins (lower panels) as markers of Golgi complex and ER, respectively. White arrows point to Golgi complex containing ATP7B-H1069Q in patient HLCs. Scale bars: 10 µm. (**b**) Immuno-electron microscopy images demonstrate distribution of ATP7B in control and patient cells. ATP7B was preferentially localized at the Golgi (white arrows) in control cells. Both cisternae of the ER (black arrows) and the Golgi membranes (white arrows) contained ATP7B in cells from patient. Scale bars: 300 nm. Graph represents % of ATP7B-associated gold particles (means ± SD) counted within ER and Golgi compartments in control and patient cells.

To assess the localization of ATP7B in patient and control HLCs, we first used confocal immunofluorescence analysis. The cells were fixed and double-labelled with an antibody against ATP7B and with antibodies against either golgin 97, to recognize the Golgi complex, or KDEL as marker of the ER. Immunofluorescence analysis showed that in control HLCs ATP7B colocalizes preferentially with golgin 97 than with KDEL (Fig. 3a). In HLCs from the patient significant amounts of ATP7B-H1069Q also were found in the Golgi and at moderate levels in the ER (Fig. 3a). This was surprising considering that when the ATP7B-H1069Q mutant is expressed in heterologous as well as in hepatic cells it resides almost exclusively in the ER, while only very small amounts of this mutant protein can be found in the distal compartments of biosynthetic pathway^9–13^. The subcellular distribution of ATP7B was therefore further investigated with immuno-gold electron microscopy (EM). As expected, in control HLCs ATP7B was preferentially localized along the membranes of the TGN, at the trans-side of the Golgi stack (Fig. 3b, white arrows). Morphometric analysis revealed that 80% of gold particles associated with ATP7B were found in the Golgi compartment of control HLCs (graph in Fig. 3). Small fraction of gold labeling was detected in the ER and could be associated with newly-synthetized pool of ATP7B wt as well as of H1069Q mutant (Fig. 3b, black arrows). Interestingly, a recent paper indicates that both wt and mutant forms of ATP7B are capable of forming a dimer^26^. This finding raises the possibility that the wt chain might help the mutant in folding and trafficking to the Golgi, resulting in low ATP7B levels in the ER of control HLCs. It would be worth testing this hypothesis in future. On the other hand, patient HLCs showed that both cisternae of the ER (Fig. 3b, black arrows) and the Golgi membranes (Fig. 3b, white arrows) contained mutant ATP7B protein. The graph in Fig. 3 shows that 35% of gold particles representing ATP7B signal were found in the Golgi and 65% in the ER. These results indicate that trafficking of ATP7B-H1069Q from the ER to the Golgi is hampered, but that the mutant maintains about 50% of the ability of the wt protein in reaching the Golgi complex.

### Patient HLCs exhibit faster degradation rates of the ATP7B protein product

**Figure 4:**
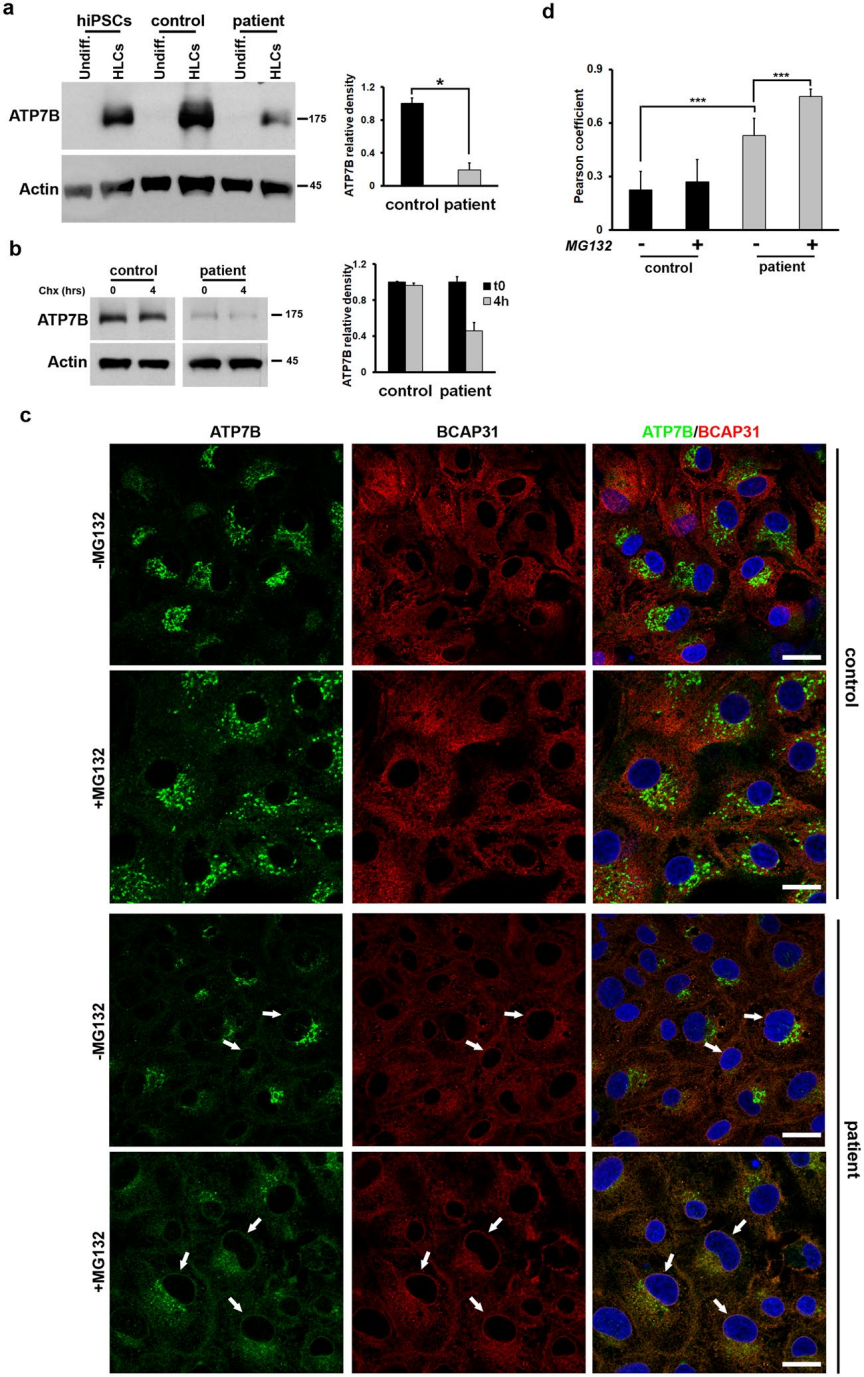
Relative accumulation and degradation of ATP7B in HLCs obtained from control and patient iPSCs. (**a**) Western blot analysis of ATP7B accumulation at steady state in undifferentiated iPSCs and corresponding differentiated HLCs. Unrelated human iPSCs were used as an additional control. The graph shows the relative expression of ATP7B in patient HLCs normalized to the level in the control HLCs. Data are reported as means ± SEM of three biological replicates. The cropped blots shown are derived from a single SDS-PAGE cut and developed for ATP7B and Actin, respectively. See Fig. S5 for full-length blots. (**b**) Western blot analysis of ATP7B accumulation in control and patient HLCs incubated for 4 h in the absence or in the presence of 100 µM Cycloheximide. The graph shows the relative expression of ATP7B at the 4 h time point normalized to the level of the 0 h time point. Data are reported as means ± SEM of three biological replicates. The cropped blots shown are derived from two SDS-PAGE run in parallel for Control and Patient samples. Both were loaded with 30 µg of cell lysate protein, cut and developed for ATP7B and Actin, respectively. See Fig. S5 for full-length blots. (**c**) Cells were treated with 30 μM MG132 for 6 h, and labeled with antibodies against ATP7B and ER resident protein BCAP31. Arrows indicate the ER (in particular nuclear envelope region), where ATP7B colocalizes with BCAP31 in patient HLCs. (**d**) Quantification of colocalization between ATP7B and BCAP31 (average ± SD; n = 40 cells) in control and patient HLCs before and after MG132 treatment (***p < 0.001; t-test). Scale bars: 6 µm.

Overexpression studies indicated that the mutant ATP7B-H1069Q accumulated in the ER is subjected to rapid degradation^9,11,13^. Considering that significant amounts of ATP7B-H1069Q move from the ER to the Golgi in HLCs, it remains unclear whether isogenic expression of ATP7B-H1069Q equally leads to fast turnover of the protein. To test this, we first compared overall ATP7B quantities in control and patient HLCs using Western blot analysis. As shown in Fig. 4, panels a and b, no ATP7B expression was detected in undifferentiated iPSCs from both control and patient HLCs, as well as from unrelated and undifferentiated human iPSCs. In contrast, strong expression was detected in the HLCs derived from the control iPSCs and the unrelated human iPSCs. Strikingly, HLCs derived from patient iPSCs showed lower expression of ATP7B-H1069Q (20% of control, graph in Fig. 4a). Furthermore, when HLCs obtained from control and patient iPSCs were incubated in the presence of the protein synthesis inhibitor cycloheximide, a clear reduction of ATP7B-H1069Q level was observed after 4 hours in patient HLCs, while no decrease was seen in the control (Fig. 4b). These results, taken together, strongly suggest that endogenously expressed ATP7B-H1069Q accumulates at lower level in patient HLCs because undergoes a faster degradation than the wt protein.

Next, we reasoned that if the mutant is degraded due to ER retention, blocking ER-associated degradation (ERAD) should increase the detectable amount of ATP7B-H1069Q in the ER. To test this, we decided to treat the HLCs with MG132, a potent, specific and reversible inhibitor of proteasome, which operates as the end point in the ERAD pathway. MG132 treatment did not induce any significant increase in ATP7B signal in the ER of control HLCs (Fig. 4c). We only noticed a limited dispersion of peri-nuclear ATP7B staining, likely due to the well-known phenomenon of Golgi fragmentation in MG132-treated cells^27^. In contrast, the ATP7B mutant was clearly detectable in the ER of patient HLCs exposed to MG132 (Fig. 4c, white arrows). Indeed, careful quantification revealed increase in colocalization of ATP7B with ER marker in patient HLCs after MG132 treatment (Fig. 4d and Supplementary Fig. S6).

Collectively, these findings indicate that the isogenic expression of ATP7B-H1069Q in patient HLCs leads to a degradation of these molecules in the ER, thus eliminating a great portion of mutant protein that can reach the Golgi complex.

### Endogenous ATP7B-H1069Q efficiently traffics in response to increasing copper

**Figure 5:**
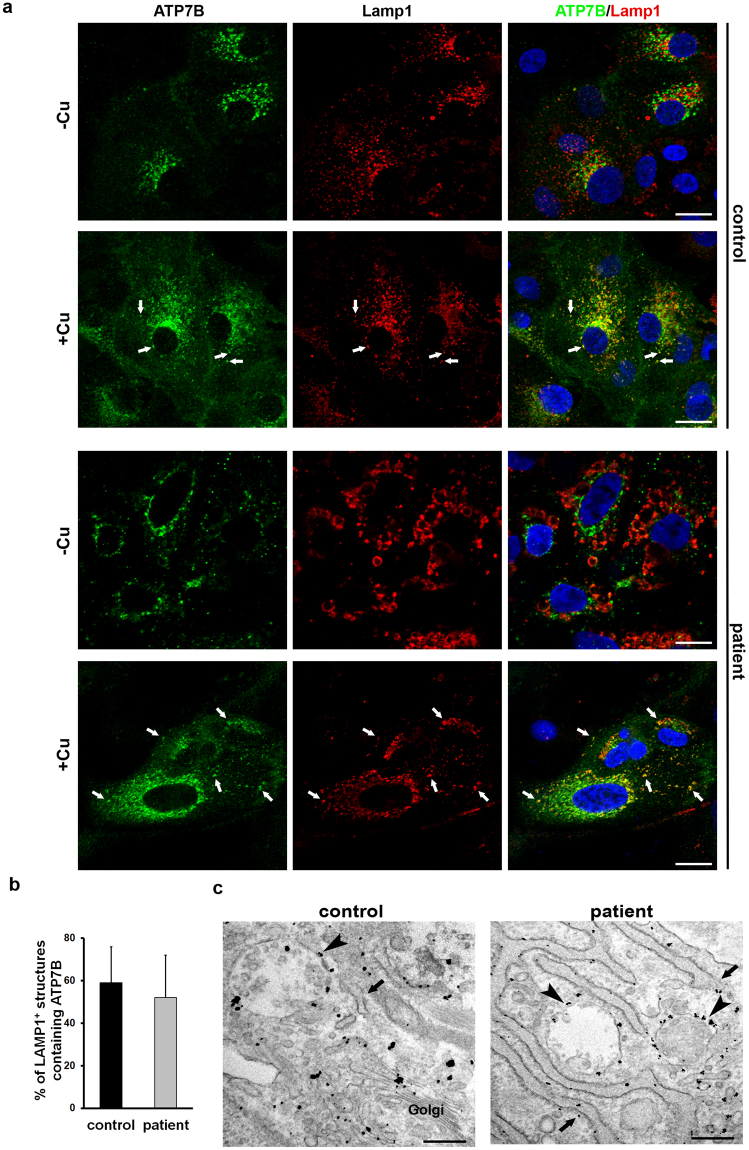
Trafficking of ATP7B in response to high copper level in HLCs obtained from control and patient iPSCs. (**a**) Cells were treated with 200 μM CuSO_4_ for 2 h and double-labeled with antibodies against ATP7B and LAMP1. Arrows indicate LAMP1-positive structures containing ATP7B both in control and patient HLCs exposed to copper. Scale bars: 5.7 µm. (**b**) The graph shows quantification of the percentage (average ± SD; n = 40 cells) of LAMP1-positive structures containing ATP7B after CuSO_4_ treatment. (**c**) Cells were treated as described in A and prepared for immuno-electron microscopy to detect distribution of endogenous ATP7B under high Cu conditions. Arrows indicate membranes of the ER. Arrowheads indicate lysosomal-like structures decorated by gold particles showing distribution of ATP7B at the external membrane of lysosomes. Scale bars: 300 nm.

The ability of ATP7B to regulate copper levels heavily relies on copper-dependent trafficking of the protein. In hepatic cells, elevated copper induces redistribution of ATP7B from the TGN to the endo/lysosomal compartments, where the transporter supports transient sequestration of excess copper into the organelle lumen^16^. Mutations that disrupt copper-dependent trafficking of ATP7B prevent elimination of toxic copper from the cytosol^14,15^. Few overexpression studies suggest that the trafficking response of ATP7B to copper can be suppressed by the H1069Q mutation^9^, while other reports indicate that when the mutant is capable to go to the Golgi it behaves like the wt protein^12,13^. Therefore, to characterize the sensitivity of endogenous ATP7B-H1069Q to copper, we exposed both control and patient HLCs to CuSO_4_ for 2 h. As expected, confocal immunofluorescence analysis showed that in control cells copper triggers a redistribution of ATP7B from the Golgi to peripheral structures containing the endo/lysosomal marker LAMP1 (Fig. 5a, arrows). Interestingly, patient HLCs exhibited identical relocation of the Golgi ATP7B pool towards endo/lysosomal compartments (Fig. 5a, arrows). Indeed, quantification revealed that upon stimulation with Cu the same percentage of LAMP1-positive structures received ATP7B in control and patient HLCs (Fig. 5b). Further immuno-EM detected ATP7B at the external membrane of multi vesicular bodies and lysosomes in both control and patient-derived HLCs exposed to copper (Fig. 5c, arrowheads). In summary, our observations suggest that ATP7B-H1069Q isogenically expressed in patient HLCs is functional in terms of copper-dependent trafficking.

Finally, all main properties of ATP7B-H1069Q were confirmed in HLCs derived from other unrelated patient. HLCs from this patient exhibited substantial amounts of ATP7B-H1069Q in the Golgi, although lower levels of ATP7B signal were detected compared to HLCs from corresponding heterozygous parental control (Supplementary Fig. S7). MG132-dependent increase of the mutant pool in the ER was also observed in this patient (Supplementary Fig. S7), indicating that endogenous ATP7B-H1069Q undergoes rapid turnover through ERAD. Lastly, Cu-dependent trafficking of ATP7B from the Golgi to lysosomes occurred with the same efficiency in the patient and control HLCs (Supplementary Fig. S7).

## Discussion

The main new result presented here is the characterization of the properties of ATP7B-H1069Q endogenously expressed in homozygous patient-derived HLCs. This characterization revealed new important features in ATP7B-H1069Q behavior, which was manifested in identical manner in two different (unrelated) patients. Moreover, this characterization was supported by the most appropriate controls, i.e. HLCs derived from the closest healthy familiar heterozygous available^28^. In addition, we describe the generation of this novel cell model that is well adapted for future work aimed at tackling this form of WD.

We investigated for the first time the dynamics (i.e. expression, turnover and copper-dependent trafficking) of ATP7B-H1069Q mutant under isogenic conditions. Almost all previous studies addressing the mechanisms behind the impact of the H1069Q mutation on ATP7B function have been performed overexpressing the protein in heterologous or hepatoma-derived cell lines^9–13^. Thus, the expression of ATP7B-H1069Q was far from isogenic conditions and the mutant protein was studied in the context of cellular proteostatic networks exhibiting different degrees of diversity respect to differentiated hepatocytes. Despite some variability, the results of these studies overall indicated that ATP7B-H1069Q protein is strongly mislocalized to the ER and has a severe trafficking impairment, mostly precluding its transport to the Golgi complex and subsequent mobilization to the endo/lysosomal compartments in response to copper excess^9–13^. Regardless of this ER retention, functional studies accredited the mutant with a significant residual copper transport activity, ranging from 20 to 70% of wt ATP7B protein according to different assays^11,18,29,30^. Thus, at a molecular level, the WD form caused by the ATP7B-H1069Q is thought to be due to a partial misfolding of the protein, which results in a massive accumulation of the mutant in the ER. As a result, the mutant fails to reach the post-Golgi sites, where ATP7B-mediated copper excretion/sequestration normally occurs.

In contrast to ATP7B-H1069Q overexpression studies, we found much more mutant protein accumulated in the Golgi complex (about 35% in the patient versus 80% in the familiar control) and, most importantly, this fraction was able to move to the endo/lysosomal compartments in the presence of excess copper as well as the transporter present in control HLCs. However, in the meantime, the total amount of ATP7B protein in patient’s HLCs was 5 times lower than in control cells, although ATP7B mRNA levels were similar. Interestingly, suppression of proteasomal degradation resulted in strong increase in ATP7B signal over the ER of patient HLCs, indicating that rapid degradation of the mutant takes place at the ER level. These last findings confirm that the H1069Q substitution destabilizes the protein^31^, most likely increasing the probability that the newly synthesized chains may undertake dead end avenues in the folding process. But the overall scenario of the molecular mechanism underlying this form of WD, and the consequent strategy to prevent its insurgence, appears now in a different perspective. Our new results in HLCs indicate that the effect of the H1069Q substitution in ATP7B is less severe than the overexpression studies have previously suggested. In HLCs, the mutant protein is fairly able to fold and to reach the TGN and post-TGN locations in response to high copper level. But, in spite of the above mentioned residual transport capacity of this mutant, the disease appears substantially due to a functional haploinsufficiency. Indeed, given the higher expression level, control HLCs locate at least 10 times more transporter in the TGN. Furthermore, assuming that ATP7B-H1069Q maintains on average 30–40% of transporter activity of the wt protein^11,18,29,30^, the patient would bear no more than 3% of the Cu-excreting capacity compared to the control.

Previous studies have reported the generation of iPSCs from WD patients with R778L and M769V genetic mutations of ATP7B^32,33^. To our knowledge this is the first report of the generation of human iPSCs from patients bearing the H1069Q ATP7B mutation. In addition, our work has obtained two advancements crucial for future applications: (i) the use of episomal plasmids instead of retro or lentivirus for reprogramming induction, thus avoiding genetic integration and the risk of changing the genetic asset of the patient cells and (ii) the use as control of the closest family member available. Previous works have used human embryonic stem cells derived from human embryo of unrelated individuals as controls. Naturally occurring genetic variation between individuals can impair the identification of disease-relevant phenotypes and can be drastically reduced by using family controls.

The availability of our model is of great importance for developing new therapeutic approaches towards WD, especially when compared to the information gained on deltaF-cystic fibrosis transmembrane conductance regulator (CFTR) mutation that cause cystic fibrosis in 90% of the patients. This CFTR mutant is rapidly turned over and retained in the ER. Over the years, molecular studies have revealed a proteostatic network that drives deltaF-CFTR degradation, while numerous screenings libraries identified both molecular targets and chemical compounds for efficient correction of CFTR^34^. Unfortunately, most of these correction strategies failed to rescue CFTR function in patient-derived cells^35^. The main reason behind this failure was that these correction strategies were developed in heterologous cellular systems overexpressing deltaF-CFTR and bearing a proteostatic network different from patient-derived cells^35^.

The HLCs described in this work: (i) have human origin, (ii) exhibit main hepatocyte features, (iii) endogenously express the ATP7B-H1069Q mutant and (iv) can be obtained from iPSCs in substantial amounts *in vitro*. This iPSCs-based strategy may be further extended in future work: patient-derived iPSCs could be extremely useful to study mechanisms behind neurologic manifestations in WD associated to H1069Q mutation of ATP7B^36^. The pluripotency of iPSCs allows them to be efficiently differentiated into neurons and opens new avenues for evaluation of the impact of this mutation in the brain where ATP7B is expressed in several neuronal cell populations^37^.

Our results demonstrate that rapid degradation is the major cause for loss of ATP7B function in H1069Q patients and indicate that new therapeutic strategies for rescuing ATP7B-H1069Q function must primarily target ER-associated degradation. On this line, the isogenic cell model described here will be instrumental for screening and validating mutant-correcting agents.

## Methods

### Informed patient consent

Informed consent to participate to this study was obtained from both the patients (indicated throughout the manuscript as #1 and #2) and from the respective familiar controls (the mother for the patient #1 and the brother for the patient #2). Written informed consent was obtained from the patients and relative familiars participating to this study also for the publication of these case reports and any accompanying images. All research was performed in accordance with relevant guidelines/regulations.

### iPSC generation and differentiation

To obtain primary fibroblasts, skin biopsies from patient and the healthy control were cut (1 mm × 3 mm) into small pieces using a scalpel blade and cultivated on cell culture dishes in fibroblast medium, consisting of DMEM supplemented with 20% FBS, 2 mM L-glutamine and 1% penicillin and streptomycin (all from Invitrogen). iPSCs were generated using the method by Okita *et al*.^24^. For iPSC differentiation into three germ layers, we used previously described methods^25,38,39^. Further methodology can be found in the Supplementary Experimental Procedures.

### Generation of HLCs from iPSCs

To generate hepatocytes from both control and patient iPSC clones, we employed an already described method that allow the efficient production of HLCs. (Highly efficient generation of human hepatocyte-like cells from induced pluripotent stem cells [26]. Briefly, iPSC clones were plated on Matrigel and cultivated for 20–22 days changing the media composition as follows: 5 days in RPMI media supplemented with B27 and 100 ng/ml of Activin A (R&D), 5 days in RPMI media supplemented with 20 ng/ml BMP4 (R&D) and 10 ng/ml bFGF (Preprotech), 5 days in RPMI-B27 supplemented with 20 ng/ml hepatocyte growth factor (HGF, Invitrogen) and finally further 5–7 days in Hepatocyte Culture Medium (Lonza) supplemented with 20 ng/ml Oncostatin M (Invitrogen).

### Preparation of cell extracts, SDS-PAGE and Western blot analysis, immunofluorescence, immuno-gold EM

These methods were used as described elsewhere^16,40^ and detailed in the Supplementary Experimental Procedures.

### Statistical analysis

The number of biological replicates of each experiment is indicated in the figure legends. The means of at least 2 independent experiments were used to calculate SEM or SD and to perform statistical analysis (when appropriate). All P values were calculated by Student’s t test.

### Data availability

All data generated or analysed during this study are available upon request. All data generated or analysed during this study are included in this published article [and its supplementary information files].

### Ethics approval and consent to participate

Authorization # 271/16 of the Ethical Committee of the University of Naples Federico II.

## Electronic supplementary material

Supplementary Material
