# Author Correction: Characterization of the most frequent ATP7B mutation causing Wilson disease in hepatocytes from patient induced pluripotent stem cells

**DOI:** 10.1038/s41598-020-65961-7

**Published:** 2020-06-23

**Authors:** Silvia Parisi, Elena V. Polishchuk, Simona Allocca, Michela Ciano, Anna Musto, Maria Gallo, Lucia Perone, Giusy Ranucci, Raffaele Iorio, Roman S. Polishchuk, Stefano Bonatti

**Affiliations:** 1 Department of Molecular Medicine and Medical Biotechnology, University of Naples Federico II, Naples, Italy; 2 Department of Translational Medical Science, Section of Pediatric, University of Naples Federico II, Naples, Italy; 3 Telethon Institute of Genetics and Medicine, Pozzuoli, Italy

Correction to: *Scientific Reports* 10.1038/s41598-018-24717-0, published online 19 April 2018

This Article contains errors. In Figure 2 F, the panels for the DAPI stain of Control #1 and Control #2 are incorrect. The correct Figure 2 appears below as Figure 1, and includes a merge of the DAPI and ATP7B images.

**Figure 1:**
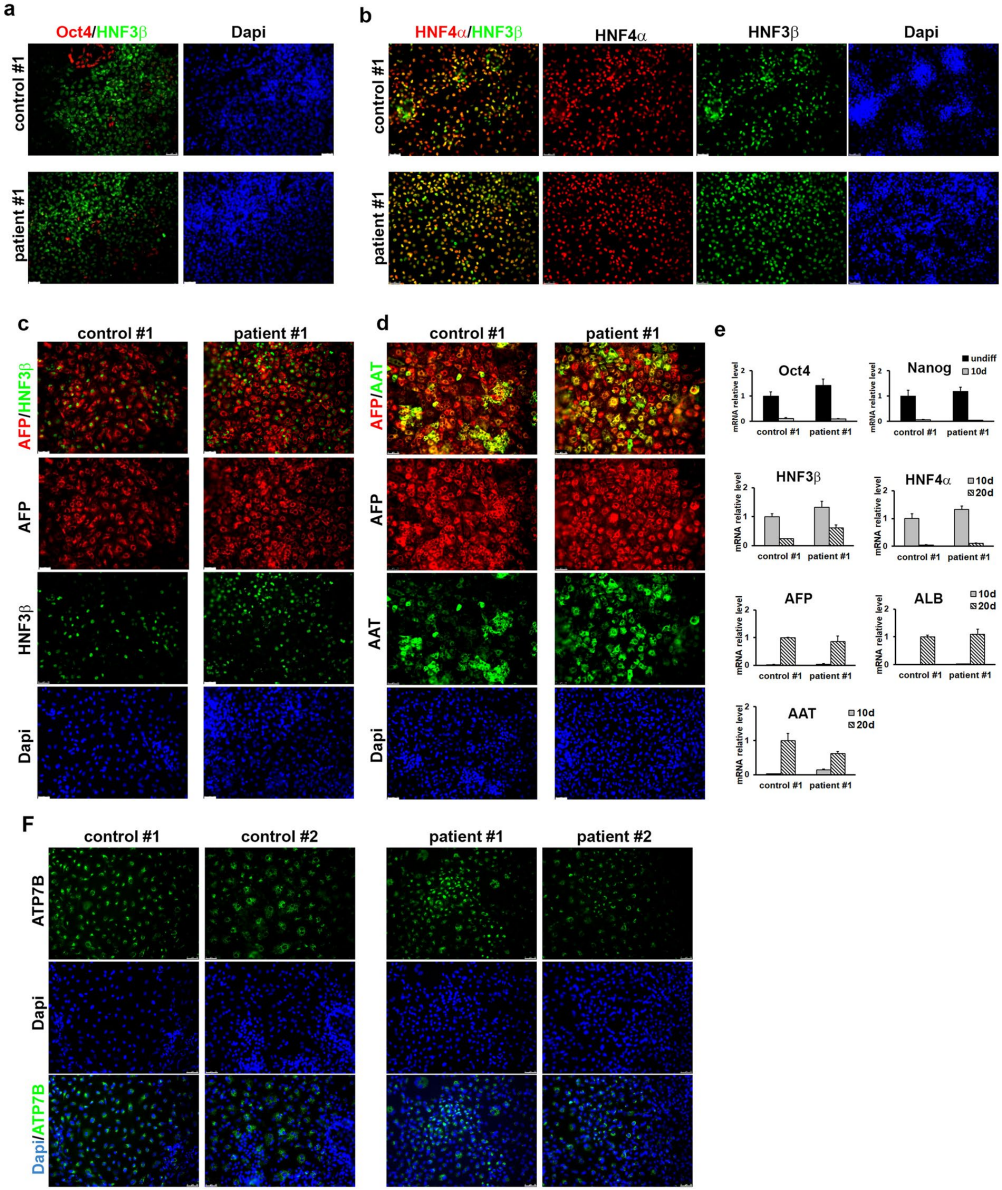
.

