# Supplementary material for: Characterization of the most frequent ATP7B mutation causing Wilson disease in hepatocytes from patient induced pluripotent stem cells
Source: Sci Rep. 2018 Apr 19;8:6247. doi: 10.1038/s41598-018-24717-0 (PMC5908878; doi:10.1038/s41598-018-24717-0)
Supplement: Supplementary file 1 — Supplementary Material [file 41598_2018_24717_MOESM1_ESM.pdf]

Supplementary Material

Characterization of the most frequent ATP7B mutation causing Wilson disease in hepatocytes from patient induced pluripotent stem cells

Silvia Parisi[1*], Elena V. Polishchuk[3], Simona Allocca[1], Michela Ciano[1], Anna Musto[1], Maria Gallo[1], Lucia Perone[3], Giusy Ranucci[2], Raffaele Iorio[2], Roman S. Polishchuk[3*], Stefano Bonatti[1*]

[1] Department of Molecular Medicine and Medical Biotechnology, and [2]Department of Translational Medical Science, Section of Pediatric, University of Naples Federico II, Naples, Italy; [3]Telethon Institute of Genetics and Medicine, Pozzuoli, Italy.

Corresponding authors:
Silvia Parisi, Department of Molecular Medicine and Medical Biotechnology, via S. Pansini 5, 80131 Naples, Italy; tel +39-081-7463155 fax +39-081-7463205;
silvia.parisi@unina.it
Stefano Bonatti, Silvia Parisi, Department of Molecular Medicine and Medical Biotechnology, via S. Pansini 5, 80131 Naples, Italy; tel +39-081-7463200 fax +39-081-7463205;
bonatti@unina.it
Roman Polishchuk, Telethon Institute of Genetics and Medicine (TIGEM), via Campi Flegrei 34, Pozzuoli, Naples, 80078 Italy; tel +39-081-19230636; polish@tigem.it

THE PATIENTS AND THE FAMILIAR CONTROLS

Patient # 1

The boy was born at term to healthy-looking consanguineous (second cousins) parents from a district of Naples. Perinatal period had been uneventful. At the age of 7 years, the patient was referred to our Liver Unit for increased serum levels of aminotransferases (AST 112 UI/L; ALT 252 UI/L) and gamma-glutamyl transferase (GGT 70 UI/L) identified in the context of a check-up for asthenia. On admission, the patient looked well; physical examination showed hepatomegaly. There was no history of hepatotoxic drug intake or parenteral exposure to blood products or illegal

substances. The patient did not complain of itching and had no clinical signs of genetic disorders. Growth and neurologic development were normal. Infections, autoimmune hepatitis, celiac disease, thyroid disorders, biliary system disease, cystic fibrosis and myopathy were ruled out.

Wilson disease diagnosis was based on low serum levels of ceruloplasmin, increased basal and after penicillamine urinary copper excretion and genetic analysis (Table 1). Diagnosis was confirmed by DNA sequencing of the entire family, that indicated the patient as homozygous for the H1069Q mutation of ATP7B, and the mother heterozygous *wt/H1069Q* (22). The patient was treated with zinc therapy as first line. A progressive improvement in copper metabolism parameters (urinary copper excretion < 75 mcg/24 h; urinary zinc excretion > 2 g/24 h) was observed in the following 12 months, with a normalization of aminotransferase by 18 months of treatment. The patient remained asymptomatic throughout the entire period of observation and ALT levels persisted normal. Currently he is 18 years old and has normal serum level of aminotransferases.

Supplemental table S1. Patient # 1 clinical and laboratory data at diagnosis

	Patient
Sex	M
Year of birth	1999
Age at onset (years)	8
Age at diagnosis (years)	8
Neurologic syntoms	-
Psychiatric symptoms	-
Hepatomegaly	+
Splenomegaly	-
Kayser-Fleischer (KF) rings	-
Laboratory	
Ceruloplasmin (mg/dL)	**19**
Aspartate aminotransferase (AST) (UI/L)	**112**
Alanine aminotransferase (ALT) (UI/L)	**252**
Gamma-glutamyl transferase (GGT) (UI/L)	**70**

Serum copper (mcg/dl)	**<40**
Urine Cu basal (mcg/24 h)	**60**
Urine Cu after penicillamine (mcg/24 h)	**2279**
Zinc	Responder
Brain MRI	Negative
Genetic analysis	**H1069Q/H1069Q**

Normal values: Ceruloplasmin: 20-60 mg/dL; AST: <50 U/L; ALT: <40 U/L; Serum copper: 100-150 g/dL; Urine copper: <40 g/24 h; Liver copper content: <50 g/g dry tissue.

Patient # 2

The patient was born at 38 weeks of gestation. Perinatal period had been uneventful. His father was affected by Parkinson's disease, the remaining family members were healthy-looking. At the age of 14 months, the patient showed infantile spasms with hypsarhythmia. He developed a drug resistant epilepsy with a neurodevelopmental delay. At the age of 13 years, his condition was complicated by micrography, walking disturbances, myoclonies, psychiatric disorders with self-aggression. At 27 years, he received a diagnosis of Wilson Disease based on low serum levels of ceruloplasmin, increased basal urinary copper excretion, neurologic symptoms and Kayser Fleisher rings (Table 1). The patient was treated with penicillamine as first line. A stabilization in copper metabolism parameters (urinary copper excretion 250-500 mcg/24 h) was observed in the following years, with complete normalization of aminotransferase by 2 years of treatment, but with the persistence of a severe neurologic deterioration with dysphagia and a complete limitation of deambulation by 14 years. At the age of 41 years, the patient was admitted at our Hospital where, on the basis of the severity of neurological symptoms and hypertransaminasemia, penicillamine was replaced with zinc acetate. On admission, brain MRI showed hyperintensity in the basal ganglia, in brainstem (pontine tegmentum and red nucleus) and in thalamus. Normalization of liver enzymes was observed in the following 12 months of zinc therapy with a significant improvement in dysarthria, dysphagia and the resumption of deambulation with reduction of synkinesia. Neurologic improvement was documented also by

transcranial magnetic stimulation studies and neuropsychological/neuropsychiatric assessment. The patient remained stable during the following 4 years and ALT levels persisted normal.

Supplementary Table S2. Patient # 2 clinical and laboratory data at diagnosis

	Patient
Sex	M
Year of birth	1972
Age at onset (years)	13
Age at diagnosis (years)	27
Neurologic syntoms	+
Psychiatric symptoms	+
Hepatomegaly	+
Splenomegaly	+
Kayser-Fleischer (KF) rings	+
Laboratory	
Ceruloplasmin (mg/dL)	**8**
Aspartate aminotransferase (AST) (UI/L)	**78**
Alanine aminotransferase (ALT) (UI/L)	**98**
Gamma-glutamyl transferase (GGT) (UI/L)	**120**
Serum copper (mcg/dl)	**<0**
Urine Cu basal (mcg/24 h)	**423**
Urine Cu after penicillamine (mcg/24 h)	**-**
Penicillamine therapy duration	14 years
Zinc therapy duration	4 years
Brain MRI	Abnormal
Genetic analysis	**H1069Q/H1069Q**

Normal values: Ceruloplasmin: 20-60 mg/dL; AST: <50 U/L; ALT: <40 U/L; Serum copper: 100-150 g/dL; Urine copper: <40 g/24 h.

Supplementary methods

iPSC generation and karyotype analysis

To generate iPSCs, 1 × 10^6 fibroblasts were transfected with a total of 1 µg of each of the following episomal plasmids: pCXLE-hOCT3/4-shp53-F, pCXLE-hSK, pCXLE-hUL (all from Addgene) (23). Electroporation was carried out using a Nucleofector™ 2b Device (Lonza) employing NHDF Nucleofector Kit (Lonza) following manufacturer's instructions. After 4 days cells were plated on mitomycin inactivated fibroblasts (feeder) and cultivated in hES medium as follow: KO-DMEM, 20% Knock-out serum replacement (KOSR), 1X Glutamax, 1X µM nonessential amino acids, 50 U/ml (penicillin and 50 mg/ml streptomicin), 100 µM 2-mercaptoethanol (all from Invitrogen) and 10 ng ml-1 bFGF (Peprotech). After 20-22 days, individual iPSC colonies were isolated and expanded for multiple passages (>5) on Matrigel (BD Biosciences) in mTeSR medium (Stem cell technologies) to remove feeder layer.

For cytological analysis iPSCs were treated with colchicine (10 µg/ml) over-night at 37 °C, followed by 20 minutes with KCl 0,56%. Then, iPSCs were fixed in methanol and acetic acid (3:1), treated with trypsin (0,01% in PBS) and stained with 2% Giemsa at pH 6.8 (GTG banding). The analysis was performed using the ECLIPSE 1000 NIKON and the GENIKON SYSTEM v 3.9.8.

Mesodermal and ectodermal differentiation of hiPSCs

For mesodermal differentiation to obtain cardiomyocytes we adapted the protocol by Zhang et al. (24). Briefly, control and patient iPSC colonies were detached from the plate using dispase and after sedimentation were resuspended in the following medium: DMEM/F12 (Invitrogen), 20% FBS (Hyclone), 1X Glutamax, 1X µM nonessential amino acids, 50 U/ml (penicillin and 50 mg/ml streptomicin), 50 µM 2-mercaptoethanol (all from Invitrogen). After 4 days, the aggregates were plated on Matrigel coated plates and 50 µg/ml of ascorbic acid (Sigma) was added to the medium. The medium was changed on alternative day for further 21 days.

For ectodermal differentiation to obtain neurons we adapted a previously used method (25). Control and patient iPSCs plated on Matrigel were induced to differentiate in RPMI supplemented with 1XN2, 50 U/ml penicillin and 50 mg/ml streptomycin (all from Invitrogen). After 7 days of differentiation 1X B27 (Invitrogen) and 1µm retinoic acid (Sigma) were added and kept for the following 7-18 days. The medium was changed every 2 days.

Immunofluorescence

hiPSCs were fixed in 4 % paraformaldehyde, permeabilized with 0.2 % TX-100 in 10 % FBS/1 % BSA in 1× PBS for 7 min at room temperature and non-specific signals were blocked in 10 % FBS/1 % BSA in 1× PBS for 30 min at room temperature. Thus, the cells were incubated with primary antibodies in 10 % FBS/1 % BSA in 1× PBS over night at 4°C. Primary antibody used are:

anti-Oct4 (1:500, Santa Cruz), anti-HNF3β (1:100, Santa Cruz), and anti-Nanog (1:500, Cell Signaling), anti-HNF4α (1:200, Abcam), βIII- tubulin (1:400, Sigma), α-actinin (1:100, Sigma), anti-α-fetoprotein (1:400, Abcam) anti-AAT (1:100, Dako) and anti-albumin (1:100, Dako). Following primary antibody incubation and washes in 1X PBS, the cells were incubated with the Alexa-Fluor 488 or 594 secondary antibodies (1:400, Molecular Probes) and nuclei were stained with Dapi (1:5000, Calbiochem). Cells were visualized with a 10×/0.30 or 20×/0.40 (dry lens) objective using an inverted microscope (DMI4000, Leica Microsystems) at room temperature in 1× PBS. The images were captured with a digital camera (DFC365 FX, Leica Microsystems) using LAS-AF software (Leica Microsystems).

RNA isolation and quantitative PCR

For quantitative PCR (qPCR), total RNA was extracted by TriSure (Bioline), and first-strand cDNA was synthesized using Mu-MLV RT (New England BioLabs) according to the manufacturer's instructions. qPCR was carried out with the QuantStudio 7 Flex (Thermo Fisher Scientific) using Fast SYBR Green PCR Master Mix (Thermo Fisher Scientific). The housekeeping GAPDH mRNA was used as an internal standard for normalization. Gene-specific primers used for amplification are listed in Supplemental Table S3. qPCR data are presented as fold changes relative to the indicated reference sample using 2DeltaCt comparative analysis.

Supplemental Table S3. Primers used for qPCR.

**Name***	**Forward primer**	**Reverse primer**
AFP	CAGCCACTTGTTGCCAACTC	ATAATGTCAGCCGCTCCCTC
AAT	CAGCCACTTGTTGCCAACTC	ATAATGTCAGCCGCTCCCTC
ALB	GGGGTGTGTTTCGTCGAGAT	AGGCAATCAACACCAAGGCT

ATP7B	AGAGAAGAATTCGGTGTCCGT	ATAAGATTTTCCGACTGGCCCC
HNF3b	TGCACTCGGCTTCCAGTATG	GGAGGAGTAGCCCTCGG
HNF4a	GGACATGGCCGACTACAGTG	TGATGGGGACGTGTCATTGC
Gapdh	GTCGGAGTCAACGGATTTGG	AAAAGCAGCCCTGGTGACC
Oct4	CGAGAAGGATGTGGTCCGAG	TGTGCATAGTCGCTGCTTGA
Nanog	ACCAGAACTGTGTTCTCTTCCACC	CCATTGCTATTCTTCGGCCAGTTG

*The primer name is reported as indicated in the Figures.

Preparation of cell extracts, SDS-PAGE and Western blot analysis

After 20 days of differentiation, HLCs from control and patient iPSCs were treated with or without 100mM cycloheximide (Sigma) for the indicated time period. Cells were lysed with lysis buffer containing 10 mM Tris-HCl pH 7,4, 150 mM NaCl, 1 mM EDTA pH 8, 1% Triton X-100 supplemented with cock-tail protease inhibitors (Roche Diagnostics, Indianapolis, USA) and protein concentration was determined by Bradford assay (Bio-Rad, USA). Denaturation and reduction of protein samples of SDS-PAGE was performed at 37°C for 30 minutes. For each sample, 50 ug of total protein was loaded into 8% SDS gel and blotting was performed using a 0.45 μm nitrocellulose membrane (GE Healthcare, Germany). The blots were blocked for 1 h with 5% non-fat dry milk prepared in TBST (10 mmol/L Tris (pH 8), 150 mmol/L NaCl, 0.1% Tween 20) and subsequently incubated with primary antibody for ATP7B (rabbit polyclonal, Abcam, Cambridge, UK, ab124973), Actin (rabbit polyclonal, SIGMA, Missouri, USA, A2066-100UL) or Vinculin (mouse monoclonal, Santa Cruz, USA sc-5546) as a protein loading control, for 1 h at room temperature. Blots were washed with TBST for three times and further incubated for 1 h with secondary antibodies HRP-conjugated goat anti-rabbit (Santa Cruz, USA sc-2004) and anti-mouse IgG (Santa Cruz, USA, sc-2005). Bound antibodies were detected by the ECL system (Santa Cruz, USA, sc-2048). Densitometric analysis was performed using ImageJ 1.47v software.

Confocal immunofluorescence analysis

Control and patient HLCs, seeded on m-slide 8 Well (Ibidi, Martinsried, Germany), were fixed in 4 % paraformaldehyde for 20 min, washed and permeabilized with 0.1 % TX-100 in 1× PBS for 6 min at room temperature. Non-specific signals were blocked in 10 % FBS/1 % BSA in 1× PBS (blocking solution) for 30 min at room temperature. The cells were then incubated for 1 h with following primary antibodies: rabbit anti-ATP7B (1:100, Abcam, Cambridge, UK ab124973), rat anti-ATP7B (1:100, generous gift of S. Lutsenko, John Hopkins University, Baltimore, ND, USA), rabbit anti-BCAP31 (1:100, Proteintech, Manchester, UK, ab 11200-1-AP), mouse monoclonal anti-Golgin-97 (1:400, Invitrogen, Oregon, USA abA21270), mouse monoclonal anti-KDEL (1:200, Enzo Life Sciences, New York, USA, ab ADI-SPA-827-D), anti AAT (1:100, Dako, Agilent Technologies, Santa Clara, CA, USA, ab A0012) and VAP-A (1:500, provided by M.A. De Matteis, TIGEM, Pozzuoli, Italy). The cells were then washed 3 times in PBS and incubated with the corresponding secondary antibodies conjugated with either Alexa Fluor 488 or Alexa Fluor 568 (Life Technologies, Oregon, USA) for 45 min, occasionally stained with Dapi (Sigma-Aldrich, Missouri, USA) and mounted in moviol.

Cells were visualized with a 63x Plan-Apo/1.4 NA Oil lens using LSM510 Meta or LSM710 confocal microscopes (Zeiss, Germany). Acquisition gain for ATP7B-channel was increased in the case of patient HLCs to provide sufficient brightness for analysis of the pattern of ATP7B protein (whose expression is significantly lower in patient cells).

Quantification of ATP7B with ER marker (either VAP-A or BCAP31) was performed via calculation of Pearson's coefficient using Colocalization module of Zen 2012 software (Zeiss, Germany). Quantification of the percentage of LAMP1 structures containing ATP7B was executed with Particle Analysis Macro of open source ImageJ software.

Immuno-electron microscopy.

For pre-embedding immuno-electron microscopy HLCs were fixed, permeabilized and labeled as described previously (16). Briefly, the cells were fixed with mixture of 4%PFA and 0.05% glutaraldehyde in 0.2 M HEPES for 15 min and with 4%PFA alone for 30 min, followed by incubation with blocking/permeabilizing solution: 0.5% bovine serum albumin (BSA), 0.1% saponin, 50 mM NH_4Cl in PBS for 20-30 min. Primary anti-ATP7B antibody (Abcam, Cambridge, UK ab124973) and 1.4nm gold-

conjugated Fab' fragment of anti-rabbit IgGs (Nanoprobes, Yaphank, NY, USA) were diluted in blocking/permeabilizing solution and added to the cells overnight or for 2h respectively. GoldEnhance™ EM kit (Nanoprobes, Yaphank, NY, USA) was used to enhance ultrasmall gold particles. Then cells were scraped, pelleted, post-fixed in OsO_4 and uranyl acetate and embedded in Epon. Thin plastic sections were cut using Leica EM UC7 ultramicrotome (Leica Mycrosystems, Vienna, Austria). EM images were acquired from thin sections using a FEI Tecnai G2 Spirit BioTwin electron microscope (FEI, Eindhoven, Netherlands) equipped with a VELETTA CCD digital camera (Soft Imaging Systems GmbH, Munster, Germany). Quantification of ATP7B-associated gold particles was done using iTEM software (Soft Imaging Systems GmbH, Munster, Germany).

SUPPLEMENTARY FIGURES

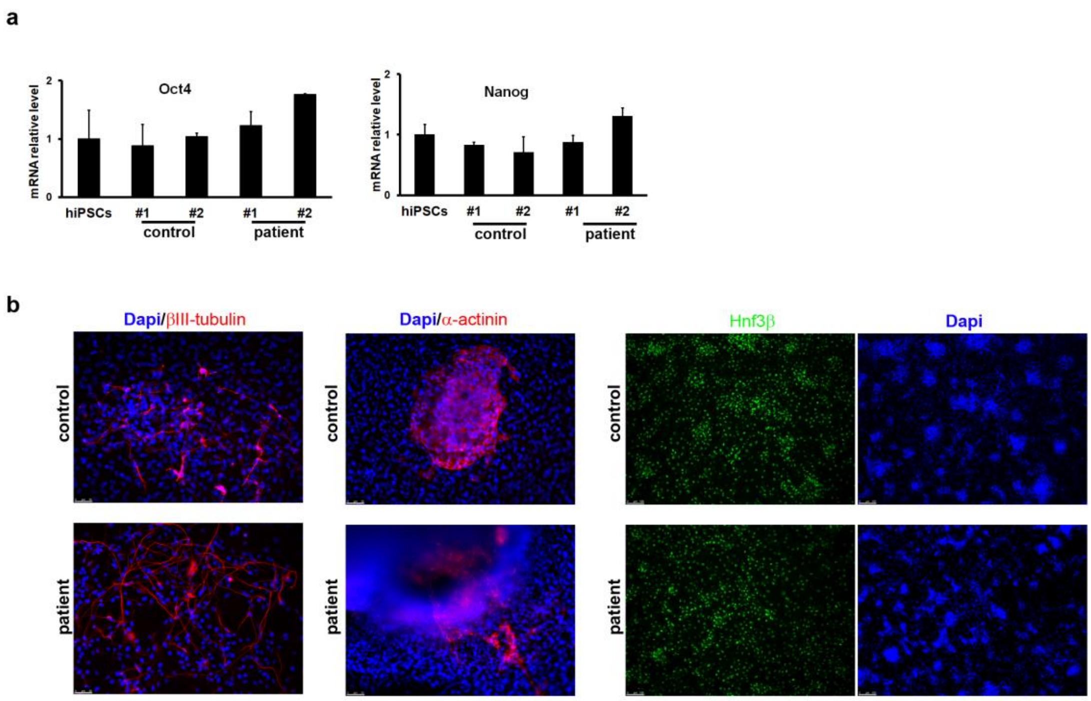

Supplementary Fig. 1: Characterization of control and patient iPSCs. (a) After generation of iPSCs, the two control and patient selected clones were adapted to grow without feeder layer and then the maintenance of the stemness phenotype was

assessed by quantitative PCR measuring the pluripotent markers Oct4 and Nanog. Unrelated human iPSCs were used as terms of comparison. The data are expressed as means (n=2) ± SEM of the fold changes relative to the unrelated hiPSCs. (b) The pluripotent potential of control and patient iPSCs was measured by immunofluorescence using neural (b3-tubulin), cardiac (a-1 actinin) and endodermal (HNF3b) markers of the three germ layers. Scale bars: 50 μm (left panels), 100 μm (right panels).

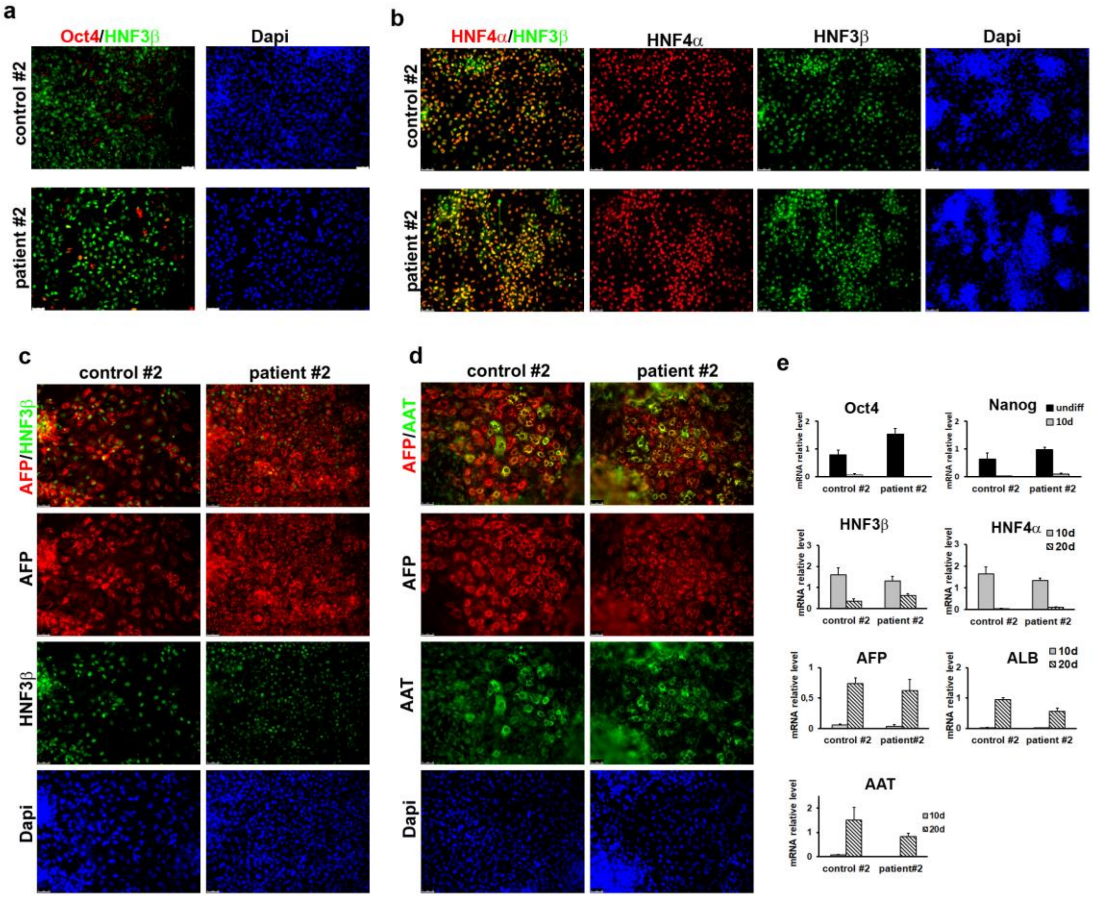

Supplementary Fig. 2: Characterization of HLC induction from control and patient iPSC clones. (a) The induction toward HLC phenotype was measured after 5 days of differentiation in control and patient iPSC clones n. 2. The disappearance of Oct4 positive cells (undifferentiated cells) and the strong induction of HNF3β (endodermal marker) positive cells indicated the achievement of endodermal phenotype. (b) After 10 days from HLC induction, the co-expression of HNF4α

(hepatic progenitor marker) and HNF3β showed the hepatic specification of endodermal cells in both control and patient cells. (c) The differentiation of control and patient cells into hepatic precursors was assessed at 15 days of differentiation by immunofluorescence for the hepatic cells marker AFP and HNF3β. (d) The achievement of the mature hepatic phenotype was evaluated at 20 days analyzing the presence of the functional hepatic marker AAT co-expressed with AFP. Scale bars: 50 µm. (e) The differentiation into HLCs was evaluated at the indicated time points by real time PCR measuring the loss of the undifferentiated phenotype (Oct4 and Nanog) and the appearance of markers of the three hepatic developmental stages: definitive endoderm (HNF3β), hepatic progenitors (HNF3β, HNF4α, AFP) and hepatic-like cells (AFP, AAT). The graphs represent the mRNA relative expression of the indicated marker normalized to the level in the control clone #1 shown in Fig. 2. Data are reported as means ± SEM of at least three biological replicates.

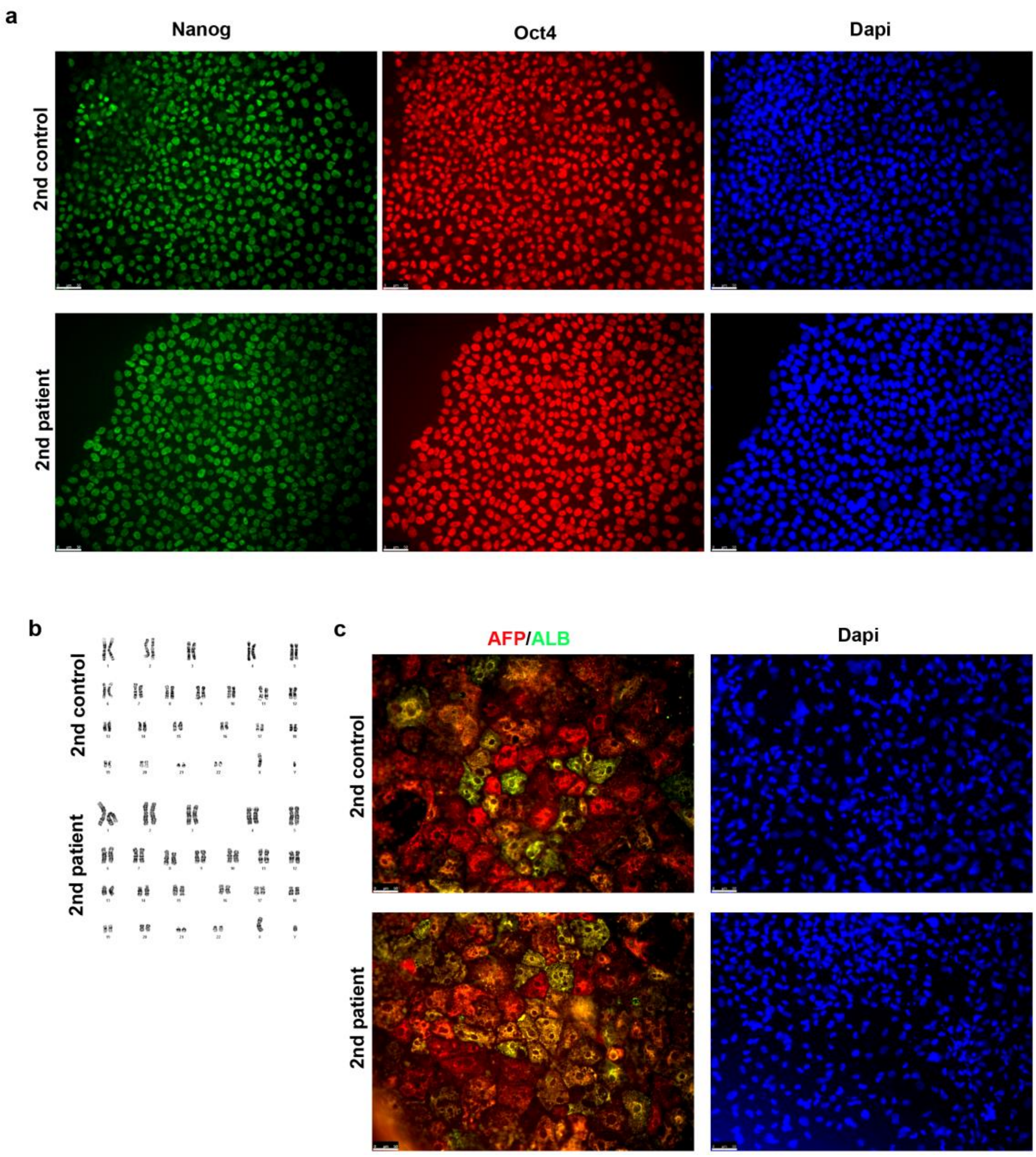

Supplementary Fig. 3. Generation and differentiation of iPSCs from a second family of WD patient and control.

(a) Human fibroblasts from 2nd control (wt/H1069Q) and 2nd patient (H1069Q/H1069Q) were reprogrammed using the protocol described in Methods section. One iPSC clone from patient and one from the control were selected for further study and adapted to grow without feeder layer. After 10 passages on matrigel the cells were stained with antibodies against the stemness markers Oct4 and Nanog to assess the mainteinance of the undifferentiated state. Nuclei are stained with DAPI. Scale bars: 50 µm. (b) Karyotype analysis of the iPSC clones from control and patient

shown in (a). No aberration in chromosomes number and structure are detectable. (c) The 2nd control and 2nd patient iPSC clones shown in panel (a) were induced to differentiate into HLCs and after 20 days the achievement of full hepatic differentiation was evaluated analyzing the presence of the hepatic marker ALB (albumin) co-expressed with AFP. Scale bars: 50 μm.

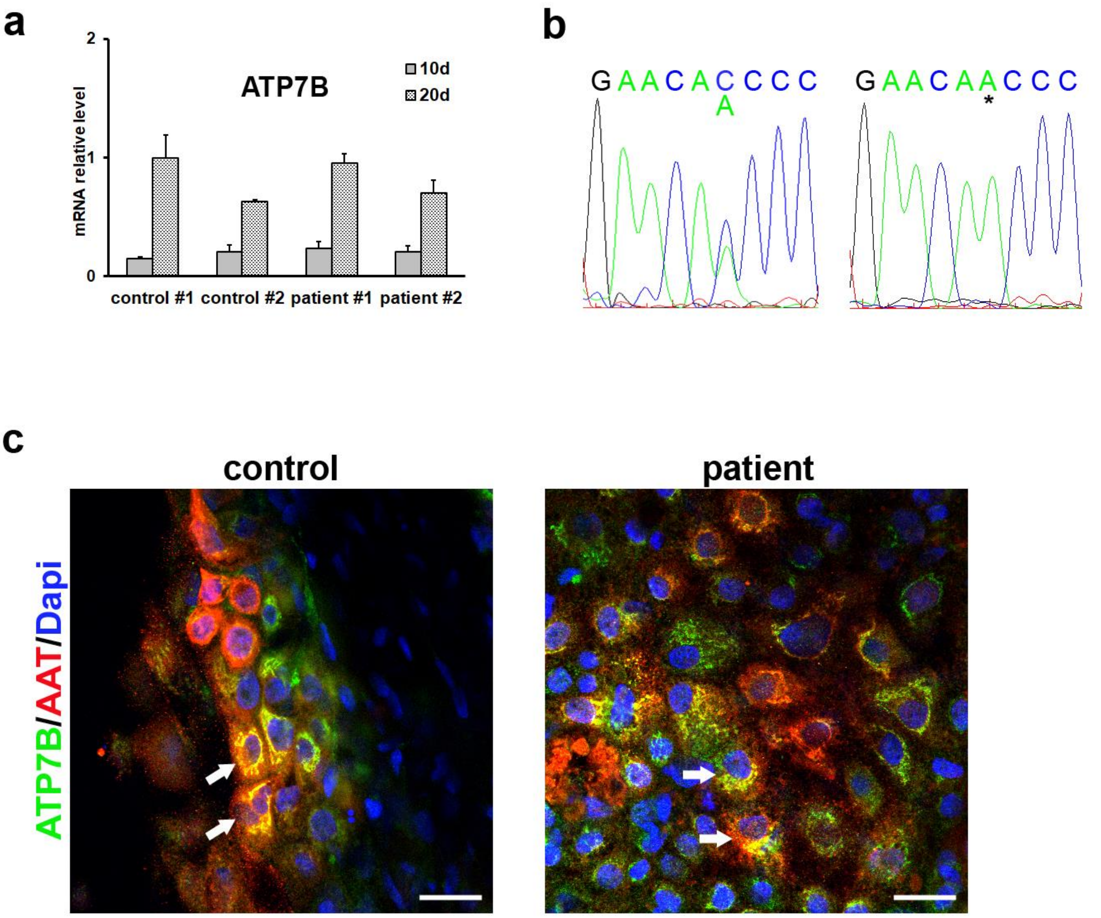

Supplementary Fig. 4: ATP7B expression in HLCs obtained from the control and patient iPSC clones. (a) The expression of ATP7B mRNA was evaluated by quantitative real time PCR at two different time points of HLC differentiation as indicated. The graph indicates that ATP7B is induced during hepatic differentiation and its expression was comparable in HLCs from patient and control iPSCs. Data are expressed as means (n=3) ± SEM of the fold changes relative to the control clone #1. (b) The heterozygosity and homozygosity of ATP7B mutation in control and patient HLCs was verified by sequencing the amplicon obtained after RT-PCR of the mRNAs extracted from iPSC clones differentiated for 20 days. (c) Confocal

immunofluorescent images of control and patient HLC cells co-expressing ATP7B and the hepatocyte functional marker AAT (white arrow). Scale bars 6 µm.

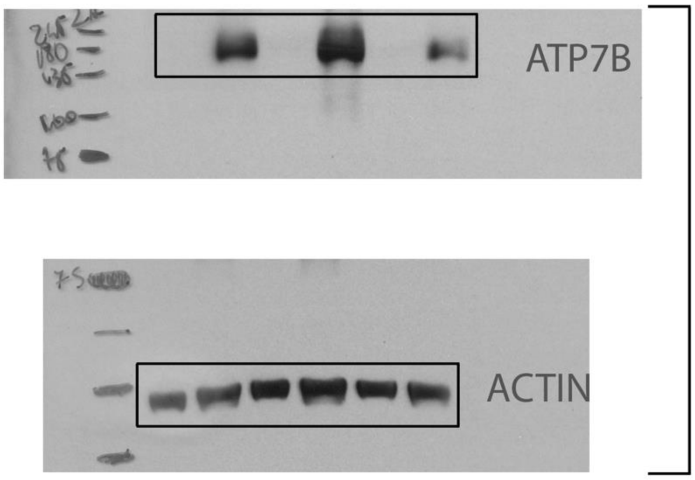

Fig.4A

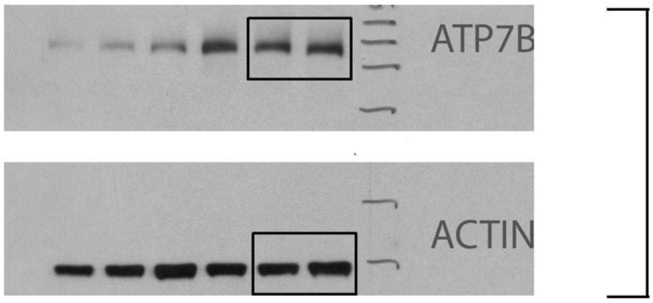

Fig.4B left panels

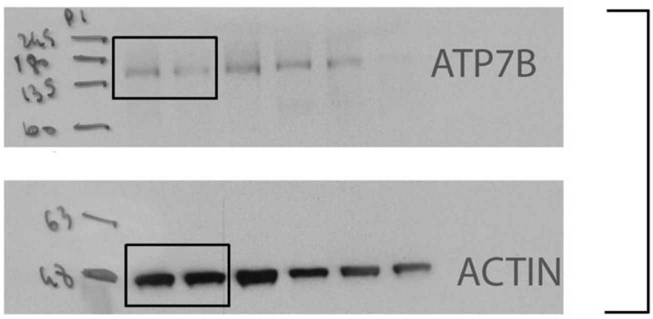

Fig.4B right panels

Supplementary Fig. 5. Full-lenght blots shown in Fig. 4, panels a and b.

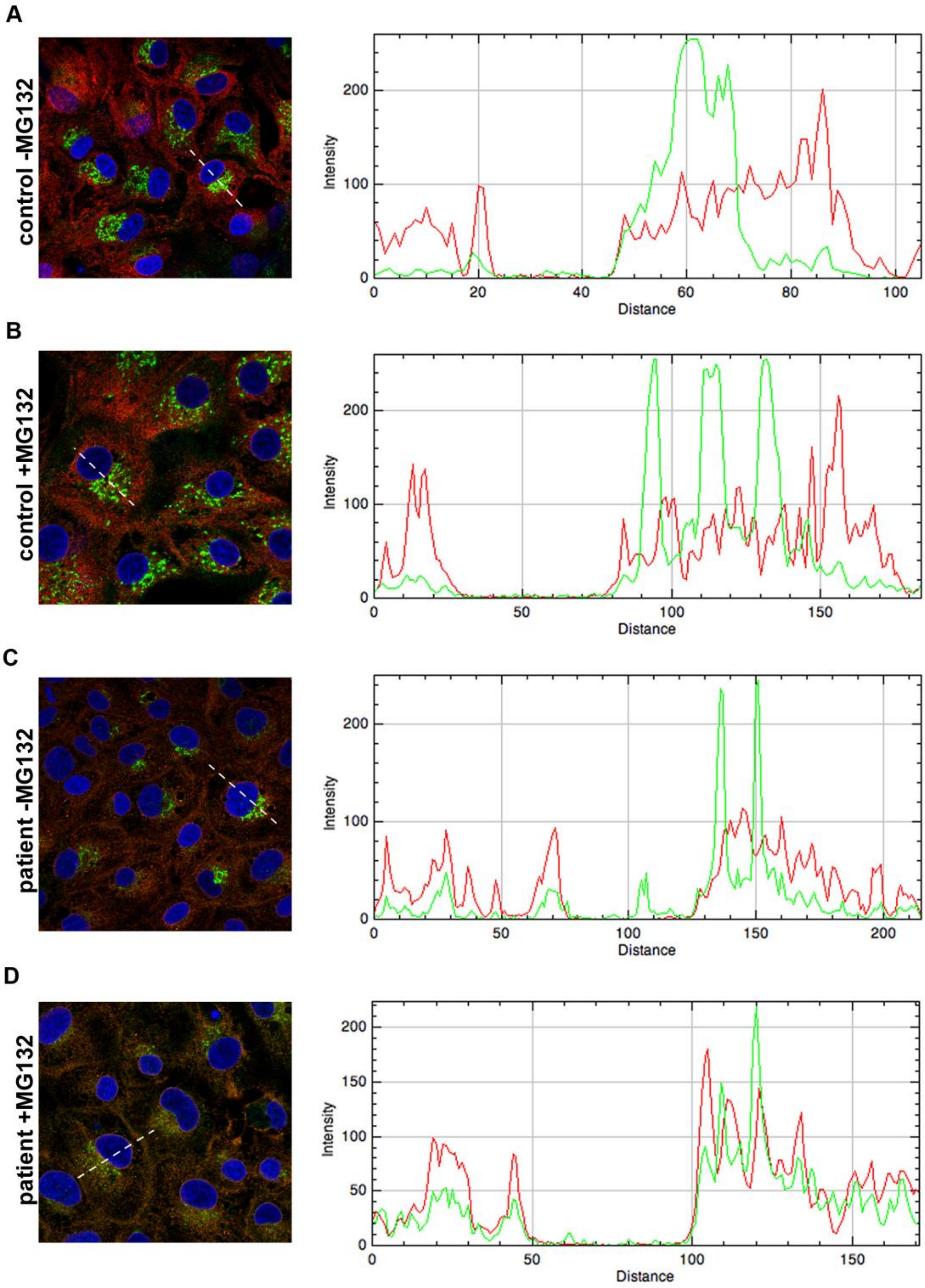

Supplementary Fig. 6. Correlation of ATP7B and BCAP31 signals increases in patient HLCs upon MG132 treatment.

The confocal images of control (A, B) and patient (C, D) HLCs correspond to the panels shown in Fig. 4C. Intensity plots for ATP7B (green) and BCAP31 (red) signals were generated using ImageJ software along the line (white dash) placed across the cell in each panel. Control HLCs (A, B) exhibit divers distribution of ATP7B and BCAP signals in line intensity plots. Red and green intensity peaks do not correspond indicating that regardless MG132 treatment ATP7B does not colocalize with ER marker BCAP31. In contrast, coincidence of red and green peaks becomes more evident in line plot from patient HLCs (C) due to more extensive ATP7B/BCAP31 colocalization. Coincidence of ATP7B and BCAP31 signals improves further upon MG132 treatment (D), indicating that treatment with MG132 leads to increase of ATP7B levels in the ER of patient HLCs.

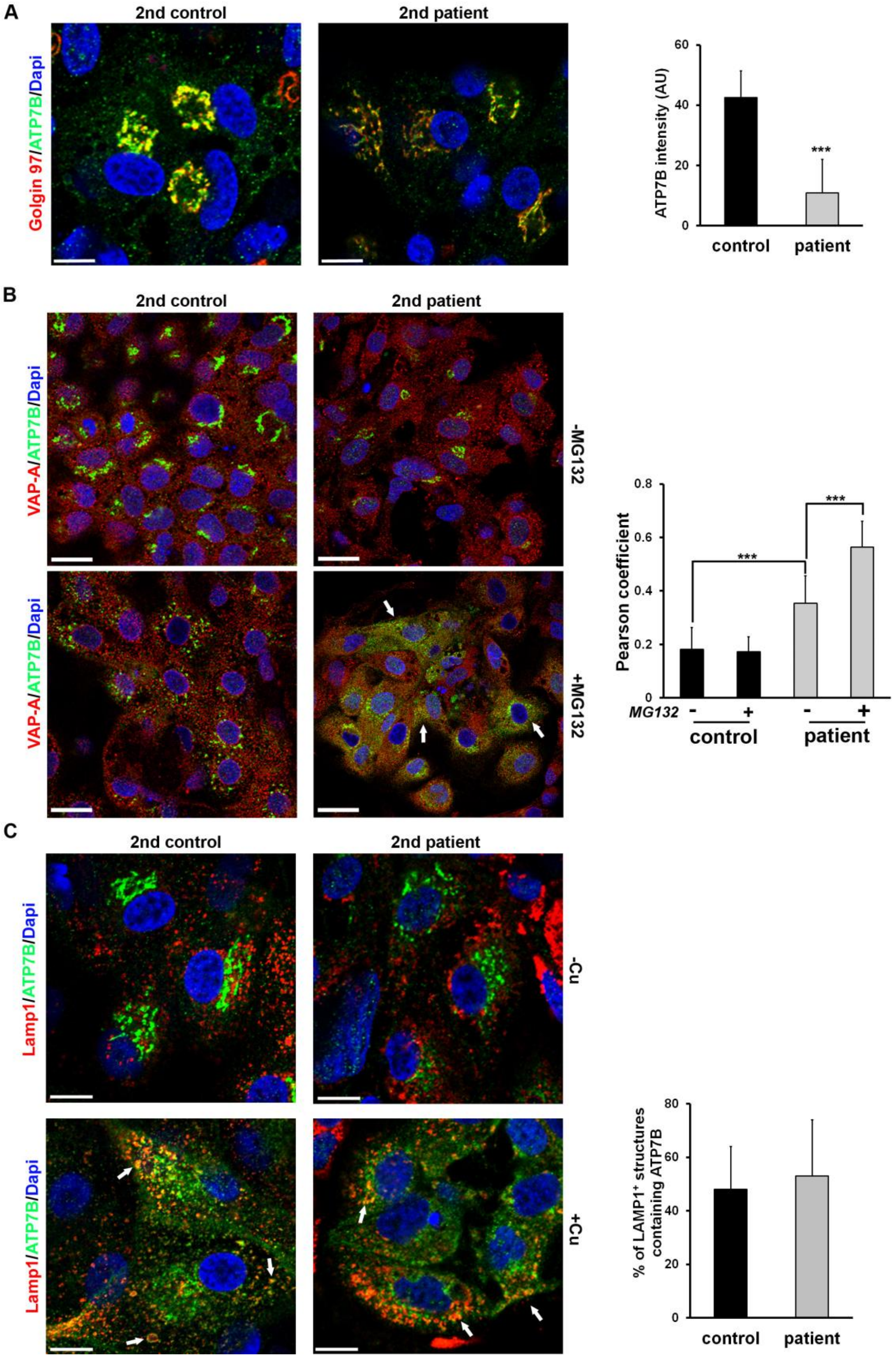

A
2nd control
2nd patient
Golgin 97/ATP7B/Dapi
ATP7B intensity (AU)
60
40
20
0
***
control
patient
B
2nd control
2nd patient
VAP-A/ATP7B/Dapi
-MG132
+MG132
Pearson coefficient
0.8
0.6
0.4
0.2
0
***
***
MG132
-
+
-
+
control
patient
C
2nd control
2nd patient
Lamp1/ATP7B/Dapi
-Cu
+Cu
% of LAMP1⁺ structures containing ATP7B
80
60
40
20
0
control
patient

Supplementary Fig. 7. Intracellular localization, MG132 effect and Cu response of ATP7B-H1069Q in HLCs from a second family of WD patient and control. (a) 2nd control and 2tnd patient iPSCs clones (the same shown in Supplementary Fig. 3) were induced to differentiate into HLCs for 20 days. Confocal images of HLCs stained for Golgin 97 (red) as marker of Golgi complex and ATP7B (green) are shown. The graph on the right shows the quantification of the intensity of ATP7B signal (in Arbitrary units) in control and patient HLCs (average ± SD; n=35 cells; *** - $p<0.001$; t-test). Scale bars: 5 µm. (b) Confocal images of 2th control and 2nd patient HLCs treated with 30 µM MG132 for 6 h and labeled with antibodies against ATP7B and VAP-A as marker of the ER. Arrows indicate the ER, where ATP7B colocalizes with VAP-A in patient HLCs. The graph on the right shows the quantification of the MG132-dependent increase in colocalization (counted as Pearson's coefficient) of ATP7B with the ER marker in patient HLCs (average ± SD; n=40 cells; *** - $p<0.001$; t-test). Scale bars: 5 µm. (c) HLCs from 2nd patient and 2nd control were treated with 200 M CuSO4 for 2 h and double-labeled with antibodies against ATP7B and LAMP1. Arrows indicate LAMP1-positive structures containing ATP7B both in 2nd control and 2nd patient cells exposed to Cu. The graph on the right shows the quantification of the percentage of LAMP1-positive structures containing ATP7B after $CuSO_4$ treatment. (average ± SD; n=20 cells; *** - $p<0.001$; t-test). Scale bars: 5µm.
